# Challenges and Prospects of New Plant Breeding Techniques for GABA Improvement in Crops: Tomato as an Example

**DOI:** 10.3389/fpls.2020.577980

**Published:** 2020-09-04

**Authors:** Pietro Gramazio, Mariko Takayama, Hiroshi Ezura

**Affiliations:** ^1^ Faculty of Life and Environmental Sciences, University of Tsukuba, Tsukuba, Japan; ^2^ Tsukuba Plant Innovation Research Center (T-PIRC), University of Tsukuba, Tsukuba, Japan

**Keywords:** γ-aminobutyric acid, GABA metabolism, tomato, health-promoting food, breeding strategies, new plant breeding techniques, CRISPR/Cas, *cis*-engineering

## Abstract

Over the last seven decades, γ-aminobutyric acid (GABA) has attracted great attention from scientists for its ubiquity in plants, animals and microorganisms and for its physiological implications as a signaling molecule involved in multiple pathways and processes. Recently, the food and pharmaceutical industries have also shown significantly increased interest in GABA, because of its great potential benefits for human health and the consumer demand for health-promoting functional compounds, resulting in the release of a plethora of GABA-enriched products. Nevertheless, many crop species accumulate appreciable GABA levels in their edible parts and could help to meet the daily recommended intake of GABA for promoting positive health effects. Therefore, plant breeders are devoting much effort into breeding elite varieties with improved GABA contents. In this regard, tomato (*Solanum lycopersicum*), the most produced and consumed vegetable worldwide and a fruit-bearing model crop, has received much consideration for its accumulation of remarkable GABA levels. Although many different strategies have been implemented, from classical crossbreeding to induced mutagenesis, new plant breeding techniques (NPBTs) have achieved the best GABA accumulation results in red ripe tomato fruits along with shedding light on GABA metabolism and gene functions. In this review, we summarize, analyze and compare all the studies that have substantially contributed to tomato GABA breeding with further discussion and proposals regarding the most recent NPBTs that could bring this process to the next level of precision and efficiency. This document also provides guidelines with which researchers of other crops might take advantage of the progress achieved in tomato for more efficient GABA breeding programs.

## Introduction

Consumer preferences are shifting towards health-promoting and functionally enriched food products that could translate into healthier lifestyles. Therefore, industry and producers are encouraged to develop and design new products to target this growing market and promote studies to test their dietary functional claims (Diana et al., 2014; Ramos-Ruiz et al., 2018). Γ-Aminobutyric acid (GABA) is widely recognized as a bioactive and functional compound thanks to a plethora of *in vitro* and *in vivo* studies reporting its beneficial effects in treating many metabolic disorders (Yoshimura et al., 2010; Yang et al., 2012). In humans, GABA functions as an inhibitory neurotransmitter (Owens and Kriegstein, 2002), and it has been reported that the intake of GABA is effective in lowering the blood pressure of hypertensive patients (Inoue et al., 2003), inducing relaxation (Abdou et al., 2006), reducing psychological stress (Nakamura et al., 2009), and shortening sleep latency (Yamatsu et al., 2016), among other health benefits. Consequently, in recent decades, the food industry has focused on releasing and developing new GABA-enriched products such as tea, yogurt, bread, cheese and fermented foods (Park and Oh, 2007; Poojary et al., 2017; Quílez and Diana, 2017). However, the GABA concentration of these products is often insufficient to confer health-promoting effects and prevent lifestyle-related disorders. Many crop plants, including fresh vegetables, have high GABA levels. Hence, plant breeders have devoted considerable efforts to developing new and improved varieties of vegetables with increased GABA contents (Lee et al., 2018). However, classical breeding is often limited by the capacity to either identify or produce (i.e., through processes such as random mutagenesis) suitable parental germplasms, apart from being time- and resource-consuming. New plant breeding techniques (NPBTs) can overcome this barrier to obtain remarkable achievements in a safer and faster way. On the other hand, GABA and other GABA genes are involved in the metabolism of many important plant species, and their modulation could be exploited to develop more well-adapted and resilient varieties.

Here, we provide an overview of the main aspects of GABA metabolism and how NPBTs could help to increase plant GABA contents using tomato as a model fruit-bearing crop.

## GABA Contents in Crop Species

After its first detection in potato tubers in 1949 (Steward et al., 1949), GABA has been found and measured in almost all economically important and model crop species, apart from bacteria, fungi and animals (Ramos-Ruiz et al., 2018). Some crops, at the harvest stage, accumulate considerable GABA levels that can substantially contribute to the daily recommended intake of GABA (10–20 mg) to generate positive health effects (Fukuwatari et al., 2001; Kazami et al., 2002; Inoue et al., 2003; Nishimura et al., 2016). The GABA content in crops varies across species and varieties and depends on a multitude of factors, such as the plant developmental stage, environmental conditions, response to biotic and abiotic stresses, and postharvest treatments (Ham et al., 2012; Kim et al., 2013; Chalorcharoenying et al., 2017) (**Table 1**). Among fresh vegetables, tomato is one of the crops that accumulates a significant amount of GABA in its edible parts, even though its content increases until the mature green stage and then rapidly decreases during ripening (Akihiro et al., 2008; Saito et al., 2008; Sánchez Pérez et al., 2011). It has been found that the GABA content in tomato also varies greatly depending on the genotype or cultivar assessed. Saito et al. (2008) reported a range of 39.6 to 102.5 mg/100 g fresh weight (FW) (0.39–1.02 mg g^−1^) of GABA in 11 fresh market cultivars and a range of 35.4 to 93.3 mg/100 g FW (0.35–0.93 mg g^−1^) in 38 processing cultivars. Higher values for processing tomatoes were reported previously by Loiudice et al. (1995), where 11 lines of San Marzano cultivars showed a range of 132 to 201 mg/100 g FW (1.32–2.01 mg g^−1^) of GABA. On the other hand, even though few genotypes have been examined, tomato wild relatives generally seem to accumulate less GABA than modern cultivars (Anan et al., 1996; Saito et al., 2008; Deewatthanawong and Watkins, 2010). This is probably due to a positive selection for the “umami” flavor, which is linked to the glutamate content.

**Table 1:** GABA content in crops.

Crop	GABA content	Reference
Apple	0.003–0.004 mg g^−1^	Deewatthanawong and Watkins, 2010; Deyman et al., 2014
Artichoke	0.011 mg g^−1^	Dosi et al., 2013
Asparagus	0.15 mg g^−1^	Zhao et al., 2007
Banana	0.023 mg g^−1^	Wang et al., 2016
Blueberry	0.079–0.089 mg g^−1^	Zhang et al., 2014; Lee et al., 2015
Broccoli	0.031 mg g^−1^	Murcia et al., 2001
Cabbage	0.032–0.071 mg g^−1^	Park et al., 2014
Carrot	0.00014 mg g^−1^	Fan et al., 1986
Cherimoya	0.005 mg g^−1^	Merodio et al., 1998
Eggplant	0.23–0.38 mg g^−1^	Mori et al., 2013
Kiwi	0.077–0.141 mg g^−1^	Macrae and Redgwell, 1992
Loquat	0.018 mg g^−1^	Cao et al., 2012
Lychee	1.7–3.5 mg g^−1^	Wu et al., 2016
Mulberry	0.86–1.86 mg g^−1^	Kim et al., 2010
Muskmelon	0.103–0.722 mg mL^−1^	Biais et al., 2010
Onion	0.001 mg g^−1^	Oh et al., 2003
Orange	0.344 mg mL−1	Zazzeroni et al., 2009
Peach	0.008 mg mL^−1^	Jia et al., 2000
Potato	0.16–0.61 mg g^−1^	Nakamura et al., 2006
Pumpkin	3.71–15.53 mg g^−1^	Qi et al., 2012
Radish	0.28 mg g^−1^	Kato et al., 2015
Raspberry	0.101–0.194 mg g^−1^	Lee et al., 2015
Spinach	0.043 mg g^−1^	Oh et al., 2003
Strawberry	0.0155–0.036 mg g^−1^	Deewatthanawong et al., 2010b
Tomato	0.35–2.01 mg g^−1^	Loiudice et al., 1995; Saito et al., 2008
Zucchini	0.026–0.040 mg g^−1^	Palma et al., 2014

## GABA Pathway in Plants

**Figure 1:**
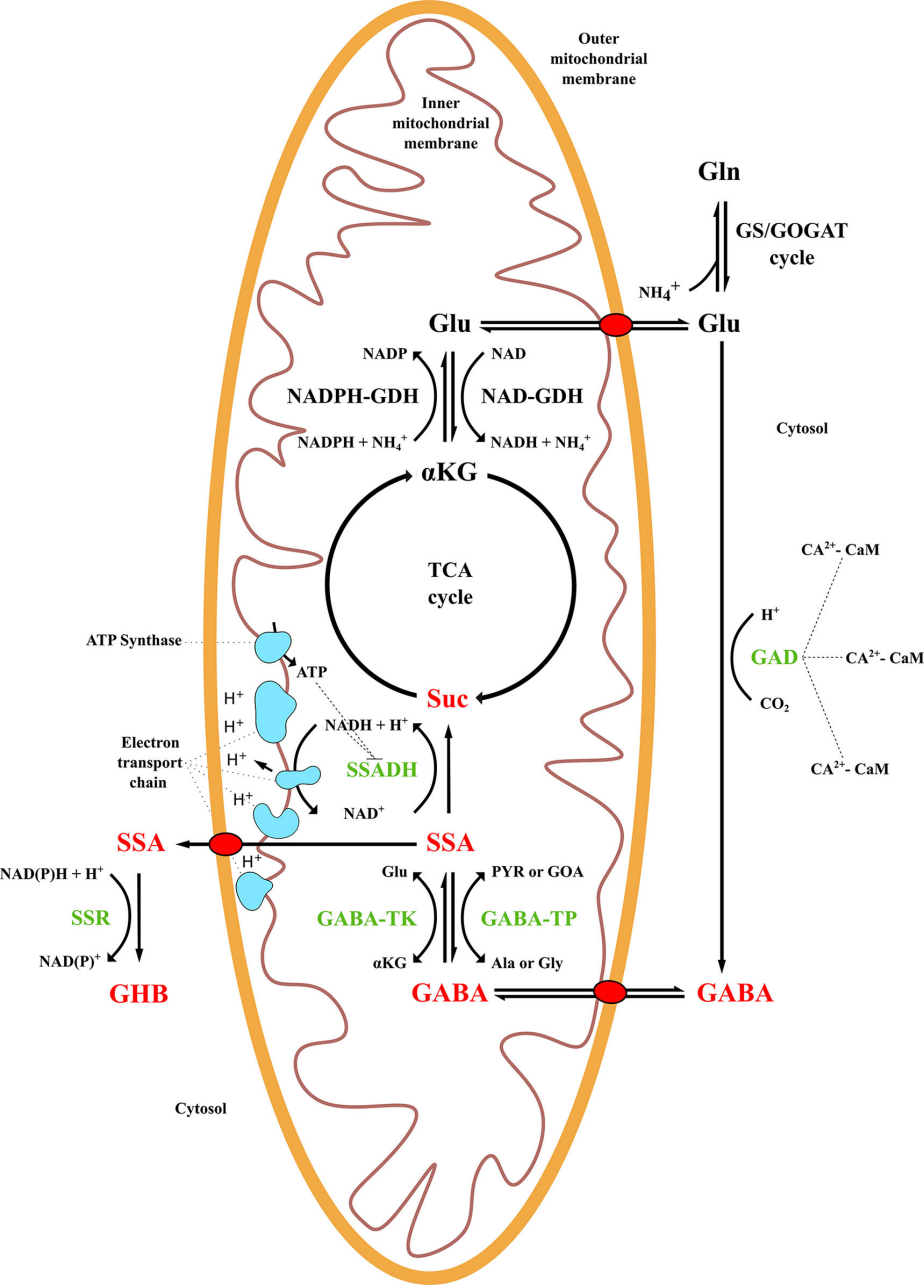
γ-Aminobutyric acid (GABA) shunt metabolism and related pathways in plant species. TCA, tricarboxylic acid cycle; GS/GOGAT, glutamine-synthetase/glutamate-synthase cycle; GAD, glutamate decarboxylase; GABA-TK, α-ketoglutarate-dependent GABA transaminase; GABA-TP, pyruvate-dependent GABA transaminase; SSADH, succinic semialdehyde dehydrogenase; SSR, succinic semialdehyde reductase; GDH, glutamate dehydrogenase; SSA, succinic semialdehyde; Suc, succinate; GHB, γ-hydroxybutyric acid; αKG, alpha-ketoglutarate; Glu, glutamate; Ala, alanine; Gly, glycine, PYR, pyruvate, GOA, glyoxylic acid.

GABA is a ubiquitous four-carbon nonproteinogenic amino acid that is widely distributed throughout the animal, plant and bacterial kingdoms. In plants, GABA is mainly metabolized *via* a short pathway called the GABA shunt, which is linked with several pathways, such as the TCA cycle (Bouché and Fromm, 2004; Shelp et al., 2017; **Figure 1**). In the cytosol, GABA is irreversibly synthesized from L-glutamate *via* the H^+^-dependent glutamate decarboxylase (GAD) enzyme, or alternatively by polyamine (putrescine and spermidine) degradation or a nonenzymatic reaction from proline under oxidative stress (Shelp et al., 2012; Signorelli et al., 2015; Yang et al., 2015). A wide range of GAD copies, which are differentially expressed according to organ types, growth stages and environmental conditions, has been identified in different plant species (Bouché and Fromm, 2004; Ramos-Ruiz et al., 2019). GAD usually presents a calcium/calmodulin (Ca^2+^/CaM) binding domain (CaMBD) at the C-terminus (30–50 amino acids) that modulates its activity in the presence of Ca^2+^ at acidic pH (Snedden et al., 1996; Gut et al., 2009). Under physiological cell conditions (pH 7.0), CaMBD inhibits GAD activity by folding its active site. Increases in cytosolic Ca^+2^ and H^+^ ions, usually as a stress response, unfold and bind CaMBD, releasing the GAD active site and stimulating its activity (Snedden et al., 1995).

The GABA shunt and the TCA cycle are connected by a transmembrane protein, GABA permease (GABA-P), that allows GABA flux from the cytosol into mitochondria (Michaeli et al., 2011). Subsequently, GABA is catabolized to succinic semialdehyde (SSA) by the transaminase enzyme GABA-T. Depending on the substrate affinity, two GABA-T enzymes can catalyze the reaction: α-ketoglutarate-dependent GABA-TK or pyruvate-dependent GABA-TP. GABA-TK receives an amino group from a-ketoglutarate and generates SSA and glutamate, while GABA-TP requires pyruvate or glyoxylate that are converted into alanine or glycine (Shimajiri et al., 2013; Trobacher et al., 2013). However, the latter has been found exclusively in plants, usually showing a higher activity than GABA-TK, while the rest of the organisms use primarily GABA-TK (Narayan and Nair, 1990; Van Cauwenberghe et al., 2002; Bartyzel et al., 2003).

Finally, SSA is catabolized in succinate, a TCA component, and NADH plus a hydrogen ion is generated from NAD^+^ by the succinic semialdehyde dehydrogenase (SSADH) enzyme (Bouché et al., 2003). Succinate and NADH are electron donors to the mitochondrial electron transport chain that produces ATP as a final outcome (Ramos-Ruiz et al., 2019). Alternatively, SSA can be converted to γ-hydroxybutyric acid (GHB) and NAD(P)^+^ in the presence of NAD(P) and a hydrogen ion by succinic semialdehyde reductase (SSR) in the cytosol or chloroplast, usually as a response to stresses (Simpson et al., 2008; Hildebrandt et al., 2015). When the NAD^+^:NADH ratio is low, the SSADH route is inhibited, as it depends on the energy balance in mitochondria, resulting in the accumulation of SSA due to the consequent GABA-T inhibition (Van Cauwenberghe and Shelp, 1999; Podlešáková et al., 2019).

## GABA Genes in Tomato

In the latest version of the tomato reference genome (Heinz 1706, version SL 4.0, Hosmani et al., 2019), five GAD genes have been annotated (Solyc11g011920, Solyc04g025530, Solyc05g054050, Solyc01g005000 and Solyc03g098240). The first GAD gene, *ERT D1* (Solyc03g098240), was predicted in 1995 from the screening of the “Ailsa Craig” tomato fruit cDNA library, showing peak expression at the fruit breaker stage and slowly declining during ripening (Gallego et al., 1995). Subsequently, from immature fruits of “MicroTom”, three GAD genes were characterized during fruit development: *SlGAD1* (Solyc03g098240, allelic to *ERD D1*), which reaches its highest expression at mature fruit stages, and *SlGAD2* (Solyc11g011920) and *SlGAD3* (Solyc01 g005000), which increase their expression during fruit development and rapidly decline during ripening (Akihiro et al., 2008). However, while *SlGAD1* did not exhibit a clear correlation with the GABA content during fruit ripening, *SlGAD2* and *SlGAD3* showed a positive correlation, suggesting a main role of the latter in GABA biosynthesis. This was confirmed by Takayama et al. (2015) by suppressing the three *SlGAD* genes through the RNAi approach, resulting in a significant GABA reduction for *SlGAD2* and *SlGAD3*-suppressed lines in mature green fruit, while *SlGAD1* GABA levels were similar to those of the wild type (WT).

In tomato, three GABA-TP genes were suggested to catabolize GABA to SSA: *SlGABA-T1* (Solyc07g043310) found in mitochondria, *SlGABA-T2* (Solyc12g006470) in the cytosol and *SlGABA-T3* (Solyc12g006450) in plastids (Akihiro et al., 2008; Clark et al., 2009). By RNAi loss-of-function analyses, Koike et al. (2013) suppressed the three *SlGABA-T* genes and observed an increase in GABA of up 9-fold in red mature fruits of *SlGABA-T1*-suppressed lines, while no significant correlation was observed between the GABA content and *SlGABA-T2* and *SlGABA-T3* expression. In light of these results and the fact that *SlGABA-T1* is expressed at a higher level than *SlGABA-T2* and *SlGABA-T3* during fruit ripening (Clark et al., 2009), *SlGABA-T1* was suggested as the major *GABA-T* gene responsible for GABA catabolism. These results were confirmed by Li et al. (2018) by a multiplex CRISPR/Cas9 system targeting the three *SlGABA-Ts*. The last steps of the GABA shunt in tomato are not yet well characterized. To date, one SSADH gene (*SlSSADH*, probably Solyc09g090700) has been identified in tomato as responsible for SSA catabolism in succinate, and it was expressed in fruits across all developmental stages even though it showed a low correlation with the GABA content (Akihiro et al., 2008). On the other hand, two SSR genes have been isolated in tomato (*SlSSR1* and *SlSSR2*, probably Solyc09g018790 and Solyc03g121720) (Akihiro et al., 2008). The expression of *SlSSR1* has been found to be slightly higher in mature red fruits than in breaker fruits, while *SlSSR2* showed the opposite expression pattern (Deewatthanawong et al., 2010a). Further studies are required to fully characterize the two alternative routes of SSA catabolism in the tomato GABA shunt and to determine how they are linked with GABA biosynthesis.

## Roles of GABA Metabolism

Across the kingdoms, a plethora of processes, functions, and pathways that are directly or indirectly involved in GABA metabolism have been identified (Seifikalhor et al., 2019). In plants, GABA plays important roles in pH and redox regulation, energy production, carbon/nitrogen balance maintenance, plant growth regulation and development, senescence, pollen germination, and fruit ripening, among other functions (Kinnersley and Turano, 2000; Bouché and Fromm, 2004; Fait et al., 2008; Renault et al., 2011; Yu et al., 2014; Carillo, 2018; Podlešáková et al., 2019). Similar to many other plant molecules, such as calcium, jasmonates, abscisic or salicylic acid, GABA rapidly accumulates in response to environmental stress (Kinnersley and Turano, 2000; Gill et al., 2016). Plants have developed highly dynamic mechanisms to face unfavorable and stressful conditions to maximize their chances to survive, modulating their responses according to the stress severity and growth stage (Podlešáková et al., 2019). Many studies on different species have reported the involvement of GABA in many abiotic stresses, such as salinity, drought, hypoxia, high/low temperature or light and nutrient deficiency/excess (Kinnersley and Turano, 2000; Renault et al., 2010; Al-Quraan et al., 2013; Espasandin et al., 2014; Wang et al., 2014; Daş et al., 2016). GABA also has inhibitory effects on biotic defenses, especially against insects and fungi (Sánchez-López et al., 2016a; Sánchez-López et al., 2016b; Scholz et al., 2017). Undoubtedly, any change affecting the genes involved in GABA metabolism, both at the sequence or expression level, could either potentiate or disrupt functions (Seifikalhor et al., 2019). In tomato, the potential roles of GABA metabolism have been investigated using, among other approaches, reverse genetics. The three *SlGAD* genes were suppressed individually and simultaneously by RNAi, demonstrating that *SlGADs* are key genes for GABA production in tomato (Takayama et al., 2015). However, the downregulation of GABA biosynthesis through SlGAD knockdown did not show a significant effect on plant growth and fruit development under stress-free conditions. In contrast, the loss-of-function of *SlGABA-T* genes resulting in drastic changes and abnormal phenotypes (Koike et al., 2013). Two out of three *SlGABA-T* mutants, *SlGABA-T1* and *SlGABA-T3*, showed severe dwarfism with a plant height half or less than that of the Micro-Tom wild type, probably due to defects in cell elongation. Additionally, *SlGABA-T1* also exhibited infertility and flower abscission, while no remarkable changes were observed in *SlGABA-T2*. Similarly, the suppression of *SlSSADH* by VIGS led to a dwarf phenotype with curled leaves, probably due to enhanced ROS accumulation (Bao et al., 2015). However, under 200 mM NaCl treatment, *SlSSADH*-suppressed plants exhibited superior salt resistance compared to that of the WT, showing a higher shoot biomass level and significantly higher chlorophyll content and photosynthesis rate. In contrast, under the same stress conditions, *SlGAD-* and *SlGABA-T*-suppressed plants showed severe salt sensitivity (Bao et al., 2015). These results suggested that tomato GABA metabolism is involved in salt stress tolerance.

More recently, Li et al. (2018), using a multiplex CRISPR/Cas9 system, targeted four GABA genes (*SlGABA-T1*, *SlGABA-T2*, *SlGABA-T3* and *SlSSADH*) and *CAT9*, a protein that transports GABA from vacuoles to mitochondria for catabolism (Snowden et al., 2015), constructing a pYLCRISPR/Cas9 plasmid with six sgRNA cassettes. Almost all edited plants with multiple mutations exhibited severe dwarfism with heights from 32.5 to 95.6% shorter than the WT. Moreover, the plants that presented higher GABA contents also showed pale green and curled compound leaves, and some of them developed a secondary axis and more leaflets with visible leaf necrosis. When analyzed by scanning electron microscopy, the leaf tissues of those mutants exhibited smaller and more compressed cells than those of the WT that were larger and stretched, confirming previous study results that suggested that GABA may be involved in plant cell elongation in vegetative tissues and leaf development regulation (Renault et al., 2011). Leaf necrosis was especially severe in *SlSSADH*-knockout lines, probably due to the increase in H_2_O_2_ levels and GHB accumulation, similar to *Arabidopsis* *SSADH*-deficient mutants (Bouché et al., 2003). Furthermore, some mutants also exhibited bud necrosis and fewer flowers, and only a few mutants set fruit, some of which were teratogenic (Li et al., 2018).

As in other plant species, GABA is involved in stress tolerance in tomato (Seifikalhor et al., 2019). Seifi et al. (2013) observed that the overactivation of the GABA shunt in the *sitiens* tomato mutant played a vital role in resistance to *Botrytis cinerea*, maintaining cell viability and slowing senescence at the site of primary invasion. The authors hypothesized that the H_2_O_2_-mediated defense *via* GABA in response to *B. cinerea* might have restricted the extent of cell death in the vicinity of the pathogen penetration sites.

More recently, it has been reported that GABA is highly involved in plant-pathogen interactions under *Ralstonia solanacearum* attack (Wang et al., 2019). Proteomic and transcriptomic profiles revealed that *SlGAD2* was downregulated, whereas *SlGABA-Ts* and *SlSSADH* were upregulated during infection. The silencing of *SlSSADH* by VIGS did not result in significant changes under *R. solanacearum* infection, while hypersusceptibility was observed for *SlGAD2*-suppressed lines, suggesting that *SlGAD2* participates in the plant defense response. However, further studies are required to understand the molecular mechanisms underlying GABA metabolism in response to pathogen attack. Thus far, it is clear that GABA levels rapidly increase under stress conditions (Shelp et al., 1999; Ramesh et al., 2017).

Recently, GABA has gained attention as a priming agent (Jalil and Ansari, 2020). Plant priming or conditioning is a promising strategy in which the plant’s physiological state is intentionally altered by natural products or synthetic chemicals to promote a more effective defense response in the case of subsequent harsher stresses (Kerchev et al., 2020). When the plant reaches the “primed” state, broad-spectrum defense is partially induced by activating defense genes, changing the proteome profile and accumulating defense compounds, among other processes, minimizing the corresponding negative effects on crop productivity (Vijayakumari et al., 2016). GABA and its synthetic isomer β-aminobutyric acid (BABA) have been reported as effective priming agents (Kerchev et al., 2020). Their application prior to pathogen infection or abiotic stress, by foliar spraying or seed soaking, triggers a similar response as endogenous GABA, modulating the latter and significantly improving the plant immune system, defense response and vigor (Malekzadeh et al., 2014; Li et al., 2017). Yang et al. (2017) reported remarkable resistance to alternaria rot (*Alternaria alternata*) after applying 100 μg ml^−1^ of exogenous GABA to tomato fruits. Although GABA did not show direct antifungal activity, it induced host-mediated resistance at the right time by activating antioxidant enzymes such as peroxidase, superoxide dismutase and catalase and reducing cell death caused by reactive oxygen species. During GABA treatment, *SlGABA-T* and *SlSSADH* were found to be upregulated. Applications of exogenous GABA at 500 and 750 μmol L^−1^ to tomato seedlings under cold stress significantly reduced electrolyte leakage, an indirect cause of chilling injury (Malekzadeh et al., 2014). GABA reduced germination under chilling stress by increasing the activity of antioxidant enzymes and the concentration of osmolytes such as proline and soluble sugars compared to those of untreated seedlings; additionally, the malondialdehyde content, an indicator of plant oxidative stress, was reduced, which results in the maintenance of membrane integrity. Similarly, 0.5 mM exogenous GABA added to a solution of 200 mM NaCl improved seedling tolerance to salt stress, enhancing the contents of antioxidant compounds such as phenolics and endogenous GABA (Çekiç, 2018). In addition to those on tomato, a plethora of studies have reported the successful usage of GABA as a priming agent in other crops, demonstrating its suitability for enhancing the innate plant immune system after stress exposure without deploying excessive energy expenses (Priya et al., 2019; Sheteiwy et al., 2019; Tarkowski et al., 2019). However, even though the success of GABA as a priming agent is compelling, the exact mechanisms of action are still puzzling.

## Pros and Cons of Increasing GABA Contents

Undoubtedly, the most important advantage to increasing the GABA content in crops and food matrices is the great potentially beneficial effect on human health, especially the antihypertensive effects (Inoue et al., 2003). Increasing the daily dietary intake of GABA might, in the long run, prevent and alleviate high blood pressure effects. In this regard, tomato is of special interest since it is the most produced and consumed vegetable worldwide and is consumed daily produce for a large part of the human population. Tomato accumulates one of the highest GABA contents among fruit and vegetables, and unlike processed food products, its taste and chemical compounds are not manipulated by additional ingredients such as sugar, salt or fat, whose excess promotes side effects. Thus, a tomato with an enhanced GABA content would benefit many people through daily dietary consumption.

However, this is not an easy task since endogenous GABA intensively accumulates from flowering to the mature green stage, being the predominant free amino acid, but rapidly declines during fruit ripening (Sorrequieta et al., 2010; Sánchez Pérez et al., 2011). Most importantly, high accumulation of GABA could provoke a severe imbalance of amino acids in cells that leads to aberrant phenotypes. Koike et al. (2013) successfully increased GABA levels by suppressing *SlGABA-T1 via* RNA interference. However, the transgenic plants showed dwarf phenotypes, with heights less than half of that of the WT, and infertility coupled with severe flower abscission. Similarly, the ﻿*Arabidopsis* GABA-T-deficient mutant *pop2* showed defectiveness in pollen tube growth and cell elongation in hypocotyls and primary roots (Palanivelu et al., 2003; Renault et al., 2011). Recently, Li et al. (2018) succeeded in increasing GABA levels up to 19-fold by a multiplex CRISPR/Cas9 system targeting the three *SlGABA-Ts* and *SlSSADH*. However, the edited plants barely set fruit, some of which were teratogenic, and showed severe dwarfism, pale green and curled compound leaves and necrosis on leaves and buds. Interestingly, Bao et al. (2015) suppressed the main genes involved in GABA metabolism by VIGS and observed a 40% reduction in GABA contents for *SlGADs*-suppressed lines and an increase of 1.5- and 2.0-fold for *SlGABA-Ts* and *SlSSADH* gene expression levels, respectively. However, only *SlSSADH*-edited plants showed defective phenotypes with curled leaves and severe dwarfism, probably due to GHB accumulation, as in the *Arabidopsis ssadh*-deficient mutant (Bouché et al., 2003).

On the other hand, attempts to increase GABA contents by manipulating *SlGADs* have resulted in less disruptive phenotypes. Takayama et al. (2015) overexpressed *SlGAD3* and observed an increase in its mRNA levels of more than 20-fold in mature green and 200-fold in red fruits that translated to an increase in the GABA content. However, those plants did not show abnormal fruits or vegetative organs. The same authors succeeded in further increasing GABA levels in red-ripe fruits compared to their previous *SlGAD3*-overexpression line by overexpressing the coding sequence of *SlGAD3*, lacking 87 nucleotides from the end in the C-terminal domain with a fruit-specific promoter (Takayama et al., 2017). Similar to other species, the removal of the GAD C-terminal domain, which is autoinhibitory, led to a significant increase in the GABA content (Baum et al., 1996; Akama and Takaiwa, 2007). Despite no morphological abnormalities observed in the new transgenic lines, defects were observed during fruit ripening. The fruits never turned red and remained orange even 30 days after the breaker stage, along with a reduction in lycopene contents and lower mRNA levels of carotenoid genes. The authors hypothesized that the removal of the C-terminal domain provoked a metabolic disturbance due to the overexpression of *SlGAD3*. At 10 days after the breaker stage, GABA accounted for up to 81% of the total free amino acids in overexpression lines compared to 6.2% in the WT, whereas low levels of aspartate and glutamate were recorded. Aberrant phenotypes were also observed in similar studies where the GAD C-terminal autoinhibitory domain was removed and significantly higher levels of GABA were accompanied by low levels of glutamate. Transgenic tobacco plants expressing a truncated GAD from petunia exhibited plant growth abnormalities coupled with reduced cell elongation in the stem (Baum et al., 1996). Similarly, dwarfism, sterility and etiolated leaves were observed in transgenic rice plants expressing a truncated *OsGAD2* (Akama and Takaiwa, 2007). These authors suggested that abnormalities could be the result of an amino acid imbalance in cells, especially low glutamate levels, due to its direct or indirect involvement in many fundamental pathways, such as gibberellin biosynthesis or posttranslational modification of cell wall proteins. Similarly, transgenic tomato fruits obtained by Bemer et al. (2012), where FUL1 and FUL2 transcription factors were simultaneously suppressed, showed orange-ripe phenotypes, two-fold higher GABA and eight-fold lower glutamate levels than the WT. The enhanced GABA fruits obtained by Takayama et al. (2017) also showed a delay in ethylene production, which peaked 10 days after the breaker stage compared with three days in the WT, and possibly a reduced ethylene sensitivity when the fruits were exposed to equivalent high levels of ethylene as the WT fruits. The authors also reported altered expression levels of transcription factors, such as TAGL1, FUL1, FUL2, RIN and ERF6, which play a major role in regulating ethylene biosynthesis, and lycopene accumulation genes, such as *ACS2*, *PSY1* and *CRTISO* (Bemer et al., 2012; Shima et al., 2013; Fujisawa et al., 2014).

Finally, overaccumulation of GABA in tomato fruit could lead to a reduction in glutamate, which is linked to the umami flavor (Yamaguchi, 1998), since glutamate is a GABA precursor. Indeed, significantly lower levels of glutamate in the high GABA tomato lines were observed, such as in *SlGABA-T1*-suppressed plants (Koike et al., 2013) and *SlGAD3ΔC*-overexpression plants (Takayama et al., 2017). Compared to the WT, the former showed an up to 11.7 times higher GABA content (13.59 vs 1.16 µmol/g FW of the WT) and 31.2% less glutamate (1.26 vs 1.83 µmol/g FW of the WT), while the latter showed an up to 18 times higher GABA content (28.56 vs 1.58 µmol/g FW of the WT) and 92.5% less glutamate (0.15 vs 2.00 µmol/g FW of the WT) in the red stage fruit. However, we have confirmed that a mild increase in GABA by 2–4 times does not dramatically affect the glutamate content, even though the effects vary among varieties (Lee et al., 2018). In addition, other molecules, such as some ribonucleotides (e.g., inosine and guanosine monophosphates), could synergistically potentiate, by a factor of 100, the umami taste in the presence of glutamate (Ninomiya, 1998; Yamaguchi, 1998; Chew et al., 2017). However, tomato flavor is the sum of complex interactions among sugars, acids and volatiles perceived between taste and olfaction, and preferences change among consumers, countries and cultures (Tieman et al., 2017).

## Strategies Deployed for Improving GABA Levels

In recent years, many strategies have been implemented to identify or breed materials to enhance GABA levels in crop species. In tomato, one of the first approaches was classical breeding through the identification of promising materials with higher GABA contents by screening the natural diversity of tomatoes for the subsequent development of new improved lines by crossing those materials with elite cultivars. Saito et al. (2008) screened 61 tomato varieties, including 38 processing and 11 fresh market cultivars, six wild species and six wild derivatives, for two years to identify naturally occurring GABA-enriched materials. However, even though an interesting variation in GABA content was found among the accessions, the results were poorly reproducible between the tested years, suggesting that GABA levels were highly influenced by the cultivation conditions. In fact, the average GABA content in red mature fruit was 50.3 mg/100 g FW in 2005 when the cultivars were screened in an open field and 66.8 mg/100 g FW in 2006 when tested in a plastic house. Nevertheless, the cultivar ‘DG03-9’ exhibited high GABA levels in both years and under saline stress (104.4 mg/100 g FW), showing its suitability to be used to breed new enhanced GABA-rich cultivars. Notably, the six wild accessions from three tomato-related species (*S. pimpinellifolium* [25.2 mg/100 g FW], *S. cheesmanii* [8.8–53.0 mg/100 g FW] and *S. lycopersicum* var. *cerasiforme* [11.0 mg/100 g FW]) exhibited a low average GABA content. Similar results were observed by Anan et al. (1996) screening accessions from *S. peruvianum* (9.8 - 10.1 mg/100 g FW), *S. pimpinellifolium* (34.2–49.7 mg/100 g FW) and *S. hirsutum* (25.5 mg/100 g FW), whereas accessions from *S. esculentum* showed appreciable GABA levels (52.4–107.7 mg/100 g FW). On the other hand, three of the six wild derivates screened by Saito et al. (2008) that reported high average GABA contents (106.4–114.7 mg/100 g FW) were bred from *S. chmielewskii*. However, so far, the highest GABA content reported from a tomato wild relative was found in *S. pennellii* at approximately 200 mg/100 g FW (Schauer et al., 2005; Takayama et al., 2017). Despite the few studies that screened for natural variations in GABA contents in tomato germplasms, which are often difficult to compare due to the tissue or stage analyzed and/or the protocol used for the GABA measurements, a wide diversity can be found across cultivated, heirloom and wild materials that can be used for GABA breeding. Classical breeding has achieved quantum leaps in crop improvement but entails some drawbacks. The development of a new variety can take up to 10 years with a classical breeding program, while that can be shortened when coupled with molecular marker selection. This makes the process time- and resource-consuming. In addition, the result is not always guaranteed when the aim is delimited to introgression of a specific alleles or traits while maintaining the genetic background and fitness of the elite variety. Furthermore, when the donor parent is a wild relative, additional challenges must be overcome, such as linkage drag of undesirable traits, crossing barriers or offspring infertility (Prohens et al., 2017).

For these reasons, alternative approaches were explored to overcome these difficulties. One of them was by screening a TILLING population generated in the background of Micro-Tom (Saito et al., 2011). However, even though approximately 4,500 EMS lines were evaluated to isolate *SlGAD3* mutant alleles, no significant mutations were found that were translated in lines with enhanced GABA levels (Ezura et al., unpublished results).

Fortunately, a wide spectrum of new technologies has emerged to sort out some of the disadvantages of conventional crossbreeding and induced mutagenesis by radiation or chemical agents. These are labeled NPBTs, which include the development of genetically modified organisms (GMOs) and genome editing. Even though there is no consensus or ultimate definition of what NPBTs are, it is an umbrella term to define several techniques that make use of a genetic modification step where the final result does not include the presence of a foreign gene (i.e., a gene that is not present in the species or cannot be obtained by traditional cross-breeding from related species) (Schaart et al., 2016). Nevertheless, every country has a different regulatory definition of NPBTs, and the list of identified NPBT technologies greatly varies from one legislation to another, being constantly under revision and discussion by policymakers. For the sake of brevity and because of the complexity and dynamic evolution of this topic, we recommend the following reviews to provide an overview of the wide range of NPBTs available and their regulation in several countries (Schaart et al., 2016; Purnhagen et al., 2018; Kleter et al., 2019; Hartung, 2020; Schiemann et al., 2020; Zhang et al., 2020).

Although GMO technologies have been used since the 1980s, first-generation GMOs cannot exactly reflect the transgene position in recipient plants. Thus, multiple transgenic events must be performed and screened to select transgenic plants with the pursued trait without undesired off-target effects (Qaim, 2020). Second- and third-generation sequencing platforms have allowed unprecedented knowledge of plant genomes and genomic regions controlling QTLs and major genes, fostering precision and speed in genome editing and breeding (Hickey et al., 2019). Since then, a new generation of mutation breeding techniques has started to target specific genomic regions, increasing specificity and reducing potential off-target effects along with saving time and resources. In fact, the greater specificity and precision of NPBTs is their main advantage over classical breeding and random mutagenesis that do not allow for specific targeting or cannot control potential additional genetic changes that might be introduced due to the linkage associated with the latter. NPBTs allow for targeting the individual genes controlling traits, allowing preservation of the genetic background of an elite variety and avoiding the undesirable effects of linkage drag.

In tomato, NPBTs have been used for understanding GABA metabolism and gene actions and investigating how to enhance GABA levels in red ripe fruits. The latter was achieved by applying two different strategies: increasing *SlGADs* activities and consequently GABA biosynthesis; and decreasing or silencing *SlGABA-Ts* activity and thus reducing GABA degradation. Although three copies of *SlGADs* and three of *SlGABA-Ts* were found in tomato, it is currently clear that *SlGAD3* and *SlGABA-T1* are mainly involved in GABA accumulation during fruit ripening. The first attempt to increase GABA content using genetic engineering was made by Koike et al. (2013), who targeted *SlGABA-T1* by an RNA interference approach under the control of the constitutive CaMV 35S in Micro-Tom. The *35S::SlGABA-T1* ^RNAi^-suppressed lines exhibited 1.3-fold (118.6 mg/100 g FW) higher GABA content than the WT at the mature green stage, 2.0-fold (126.8 mg/100 g FW) higher at the yellow stage and 6.8-fold (106.2 mg/100 g FW) higher at the mature red stage. *SlGABA-T1* suppression reduced the catabolism of GABA to SSA in the ripening stage, limiting its degradation from the peak reached at the breaker stage. While in the WT, the GABA content dropped 83.1% from the mature green to red stage, the decrease in *35S::SlGABA-T1* ^RNAi^ lines was only 3% on average. However, as reported above, those plants exhibited severe abnormalities. Nevertheless, when they replaced the 35S promoter with E8, a strong inducible promoter specific to tomato fruit ripening, to avoid the systemic suppression of *SlGABA-Ts* by the CaMV 35S promoter, the *E8::SlGABA-T1* ^RNAi^-suppressed lines showed a similar WT phenotype with no evidence of dwarfism or infertility. Although the GABA levels of *E8::SlGABA-T1* ^RNAi^ lines were similar to those of the WT at the mature green stage (71.1–87.6 mg/100 g FW), their content was 2.5-fold higher than that of WT (45.3–59.8 mg/100 g FW) at the red stage, dropping 29.0% versus 72.4% in the WT, although their GABA content was almost half of that of *35S::SlGABA-T1* ^RNAi^. These results suggested that the systemic knockout of *SlGABA-Ts*, especially *SlGABA-T1*, led to abnormal phenotypes, whereas a fruit-specific knockout, knockdown or less severe gene expression reduction produced normal phenotypes with enhanced GABA levels.

The opposite approach was attempted by Takayama et al. (2015), who overexpressed *SlGAD3* by generating transgenic plants under the control of the CaMV 35S promoter in Micro-Tom. The *OX-SlGAD3*-overexpression lines exhibited 2.7- to 3.3-fold higher GABA contents than the WT at the mature green stage and 4.0- to 5.2-fold higher GABA contents at the red stage. Despite the much higher GABA content, the *OX-SlGAD3* lines did not show significant visible phenotypic changes. The same authors, to further increase GABA levels in red-ripe fruits, overexpressed the full-length coding sequence of *SlGAD3* (*SlGAD3^OX^*) and were missing the same 87 nucleotides from the end in the C-terminal domain (*SlGAD3ΔC^OX^*), substituting CaMV35S for the fruit-specific E8 promoter and the NOS terminator for *Arabidopsis* heat shock protein 18.2 (HSP) (Takayama et al., 2017). At 10 days after the breaker stage, *SlGAD3ΔC^OX^* lines exhibited 11- to 12-fold higher GABA levels than the WT (237.1–268 mg/100 g FW) and almost double those of *SlGAD3^OX^* lines (123.7–154.6 mg/100 g FW). Although the latter displayed substantially higher mRNA levels than the previously generated *OX-SlGAD3* lines (Takayama et al., 2015), probably due to the *Arabidopsis* HSP terminator, which is more effective than the NOS terminator in enhancing mRNA expression (Kurokawa et al., 2013), their GABA levels were similar. This result suggested that even though increased mRNA expression translates into a higher GABA content, it does not linearly increase with the mRNA level, and other factors are involved in regulating GABA levels, such as the C-terminal domain. In fact, the *SlGAD3^OX^* and *SlGAD3ΔC^OX^* lines exhibited similar mRNA levels, but the GABA content was almost doubled in the latter, implying that the C-terminus also acts as an autoinhibitory domain in tomato. However, even though the lines of both constructs did not display any morphological abnormalities, probably by virtue of replacing CaMV35S for the fruit-specific E8 promoter, the *SlGAD3ΔC^OX^* lines exhibited a delay in ethylene production and ethylene sensitivity along with an orange-ripe phenotype that never turned completely red.

In light of these results, Nonaka et al. (2017) further investigated the effects of the C-terminal region in *SlGAD3* to breed an enhanced GABA line without defects, targeting the autoinhibitory domain by the CRISPR/Cas9 system. After comparing the amino acid sequence of *SlGAD* orthologs from five species for the conserved tryptophan, lysine and glutamate residues involved in CaM binding, the 37th amino acid at the C-terminal domain was selected as the target to induce a premature stop codon in *SlGAD3*. This target was different from the 29th amino acid (87 nucleotides) selected by Takayama et al. (2017). The T_1_ regenerated plants of *TG3C37* that had a stop codon at 34, 36 and 40 amino acids at the C-terminal, thus upstream of the autoinhibitory domain and close to the target at the 37th amino acid, and exhibited a higher GABA content at the red mature stage of up to 15 times more (125.73 mg/100 g FW) than that of the WT. Although some lines were slightly smaller than the WT, flowering and fruit yield were not affected, and no significant phenotypic defects were observed. Considering the absence of visible morphological and physiological abnormalities and the range of GABA levels obtained, these results were similar to those obtained by overexpressing the full coding length of *SlGAD3* (*SlGAD3^OX^*). The authors stated that even though targeted mutagenesis by CRISPR/Cas9 is as effective a strategy as the transgenic approach in increasing GABA contents, CRISPR/Cas9 could be more publicly accepted than conventional transgenesis in the near future.

The latest attempt to develop improved GABA tomato lines was made by Li et al. (2018) using a multiplex CRISPR/Cas9 vector with six gRNA cassettes to target the three *SlGABA-Ts* along with *SlSSADH* and *CAT9* that were transformed in the Ailsa Craig cultivar. The edited plants were divided into six groups based on the mutation patterns from single to quadruple mutated targets. However, only the single *SlGABA-T1* and the double *SlGABA-T1* and *SlGABA-T3* mutants set fruits due to the severe infertility of the rest of the combinations. The *SlGABA-T1* edited lines exhibited 1.43-fold higher GABA levels (102.80 mg/100 g FW) than the WT at the mature green stage and 2.95-fold higher (73.83 mg/100 g FW) levels at the red stage. Even though those fruits did not show differences in size, shape and color with the WT, the plants experienced abnormalities in leaf development and plant growth. Once again, these results demonstrated that *SlGABA-T1* is deeply involved in GABA metabolism, but its severe mRNA expression reduction by suppression or mutation leads to deficient plants not suitable for breeding.

## Advantages of the Use of NPBTs in Crop Breeding and Their Societal Acceptance

Genome editing is a technology that enables precise mutagenesis only in targeted genes. Therefore, using this technology, we can significantly reduce the labor and time required to introduce desirable mutations, which is a challenge for conventional mutagenesis techniques using chemical or irradiation treatments. This property would be a major advantage in breeding crops that have strong consumer preferences, require a large number of varieties in a single crop and have rapidly changing needs because to breed more varieties, more labor and time would be required. Tomatoes are one such example. They are consumed all over the world, and preferred shapes, colors, flavors and uses vary by region. Since genome editing technology can directly reproduce useful genetic mutations in breeding parents, we would be able to improve the traits of familiar varieties more rapidly and efficiently. In the case of improving the GABA content in tomato, genome editing would be effective not only in experimental cultivars but also in commercial cultivars. Indeed, we succeeded in reproducing the mutation that confers an increased GABA content in fruit in several commercial cultivars by applying the CRISPR/Cas9 system as used in experimental cultivars (Nonaka et al., 2017). It took only half a year to obtain null-segregant (T-DNA free) plants with homozygote mutations. Even though an additional selfing or backcross step would be needed to select elite lines as in conventional breeding, it can reduce the time to develop breeding material considerably when compared to conventional cross breeding, which takes 3–5 years.

However, frequently, NPBTs are confused or associated with the first generation of modified crops, where the use of foreign DNA to develop new and improved crops allows society and policymakers to perceive health and environmental risks associated with their cultivation and consumption. Even though three decades of GMO cultivation and dozens of studies reported no more human health risks than those posed by conventional agriculture (Nicolia et al., 2014; Zaidi et al., 2019), the general perception of GMO technologies remains negative, with large differences among countries. The emergence of genome editing technologies such as CRISPR/Cas has generated great expectations among scientists, breeding companies and the food industry regarding the possibility of changing consumer perceptions of food biotechnology. However, even though the societal acceptance of NPBTs is higher than that of the first GMO crops, currently, “food technology neophobia” still affects many consumers and not only high-income countries (Farid et al., 2020; Siegrist and Hartmann, 2020). Often, this confusion is promoted by the same lawmakers that legislatively, as in the case of the European Court of Justice, equate genome-edited crops with the first generation of GMOs, negatively influencing the public perceptions of NPBT products (Callaway, 2018; Zaidi et al., 2019). Nevertheless, there are grounds for cautious optimism due to the policy relaxation of some countries that are considering opening their markets to NPBT products such as China and Russia. Currently, in seven countries (Argentina, Brazil, Chile, Colombia, Israel, Paraguay and the US, plus India, Uruguay, Honduras, Guatemala and El Salvador, which are under consideration), genome-edited crops that do not incorporate foreign DNA are regulated as conventional plants with no additional restrictions, while Japan, Canada and Australia have stricter regulation of genome-edited products than conventional ones but less stringent regulation than that of GMOs that incorporate DNA from other species (https://crispr-gene-editing-regs-tracker.geneticliteracyproject.org/). In contrast, other countries such as New Zealand, Mexico or the EU strictly regulate NPBT products, basically banning their development and introduction.

## Alternative Prospects for GABA Improvement Using NBPTs

New NPBT approaches and techniques have been launched at a frantic pace in the last two decades, especially for genome editing. However, the first-generation of gene-editing methods, such as zinc-finger nuclease or TALEN, have been totally eclipsed by the CRISPR/Cas9 system. Since the first evidence of its powerful applications in 2012 (Jinek et al., 2012), the CRISPR/Cas9 system and its subsequent variants have become the most widely used NPBTs due to their simplicity, reliability and versatility. Many of these variants are focused on improving the genome editing process from the first monoguide CRISPR/Cas9 versions towards multiplexed and multilocus strategies (Vad-Nielsen et al., 2016; Zetsche et al., 2017), enhanced Cas enzymes and new ribonucleoprotein complexes (Bernabé-Orts et al., 2019; McCarty et al., 2020) and cleavage patterns (Komor et al., 2016; Shimatani et al., 2017), among others.

Apart from the performance of the genome editing system *per se*, interesting CRISPR/Cas9 applications and approaches have been proposed and validated in plants. One of the most promising is *cis*-regulatory region engineering (*cis*-engineering), which targets the noncoding sequences controlling gene transcription. Many examples have reported that mutations in *cis*-regulatory elements (CREs) produce significant phenotypic and morphological changes that have been selected during domestication (Meyer and Purugganan, 2013; Swinnen et al., 2016). In tomato, changes in CREs and promoter regions were translated in elongated (*SlSUN*) and larger fruits (*FW2.2*, *FW3.2*, *SlWUS*) or those with improved β-carotene contents (*Slcys-B*) (van der Knaap et al., 2014; Li et al., 2020) as just a few relevant examples. However, despite the great potential of this approach, currently, the vast majority of CRISPR/Cas9 studies focus on targeting coding sequences for null allele editing or controlling transcription by activation (CRISPRa) or inhibition (CRISPRi), and only 15 studies so far have reported successful applications of *cis*-engineering (Li et al., 2020). One of them was successfully conducted in tomato by *cis*-engineering the promoters of genes controlling fruit size, inflorescence branching and plant architecture, achieving multiple *cis*-regulatory alleles with a continuum of quantitative variation (Rodríguez-Leal et al., 2017). The scarcity of *cis*-engineering studies might be due to the lack of information on CRE functions and the regulatory complexity and redundancy of transcriptional control. Additionally, in contrast to targeting the coding regions that could produce substantial and pleiotropic effects, the effects of *cis*-regulatory allele targeting are often more subtle and discrete (Wittkopp and Kalay, 2012).

**Figure 2:**
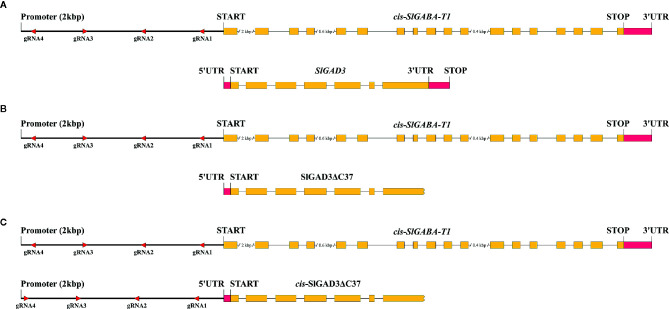
*Cis*-engineering approaches of *SlGABA-T1* and *SlGAD3* promoters to enhance the GABA content in tomato. **(A)** *Cis*-engineering of the promoter region of *SlGABA-T1* while *SlGAD3* remained the wild type to observe the effects of *SlGABA-T1* CRE editing. **(B)** *Cis*-engineering the promoter regions of *SlGABA-T1* of the SlGAD3ΔC37 GABA improved line to exploit the potential synergistic effects of the *cis*-engineering and coding sequence targeting approaches. **(C)** *Cis*-engineering of the promoter regions of *SlGABA-T1* and SlGAD3ΔC37 to maximize GABA accumulation.

Perfect examples of detrimental pleiotropic effects in GABA studies were reported by Koike et al. (2013) and Li et al. (2018) who suppressed and edited, respectively, the coding sequences of *SlGABA-Ts*. In both studies, the regenerated plants showed multiple abnormalities, making them useless for breeding despite the higher GABA levels achieved. To overcome these drawbacks, the authors of this review are currently *cis*-engineering the promoter region of *SlGABA-T1* (**Figure 2**). The aim of this study was to modulate *SlGABA-T1* gene expression to achieve the best balance between reduced enzyme activity and a higher GABA content without detrimental pleiotropic effects that cause plant abnormalities. This can be theoretically achieved by producing many *cis*-engineering promoter alleles that exhibit a continuum of gene expression and CRE combinations. However, like most plant species, almost no knowledge has been generated regarding the structure of promoters and other regulatory sequences in tomato, and no prior information is currently available for CREs and their interactions in the promoter of *SlGABA-T1*. Even though prior knowledge would facilitate and speed the process, *cis*-engineering could be implemented if a multiplexed CRISPR/Cas9 system was developed (Li et al., 2020). For this, we assembled a vector containing four gRNAs that was designed within a region 2 kbp upstream of the *SlGABA-T1* start codon and transformed in Micro-Tom (**Figure 2A**). Furthermore, the same vector was also used to transform the high GABA line SlGAD3ΔC37 developed by Nonaka et al. (2017) to exploit the potential synergistic effects between the *cis*-engineering promoters in *SlGABA-T1* alleles and *SlGAD3* without the autoinhibitory domain (**Figure 2B**). A complementary approach could also involve *cis*-engineering the promoter of SlGAD3ΔC37, combining the effects of removing the autoinhibitory domain and modulating the gene expression level by *cis*-engineering (**Figure 2C**). Apart from achieving improved GABA lines, this study may provide useful information on GABA gene promoters that will open the path to future strategies that are currently unavailable.

For example, if CREs and their interactions with regulatory elements are sufficiently characterized, the multiplex unspecific gRNA approach could be replaced by site-specific promoter editing. A step further in this direction would be the upgraded precision from genome editing to base editing or the groundbreaking prime editing strategy that has recently been demonstrated to be feasible in higher plants (Rees and Liu, 2018; Anzalone et al., 2019; Lin et al., 2020).

However, other NPBT strategies could be more challenging to implement for GABA breeding until their technical limitations and low efficiency are overcome. Among those, a fascinating approach is promoter insertion or promoter swapping that allows introduction or replacement of CREs or entire promoters. However, these approaches rely on the homology-directed repair (HDR) pathway, which has exhibited low efficiency in plants (Chen et al., 2019). Nevertheless, in tomato Čermák et al. (2015) succeeded in inserting a 35S promoter upstream of the gene controlling anthocyanin biosynthesis (ANT1), resulting in enhanced anthocyanin accumulation.

## Final Remarks

The ubiquity of GABA across the kingdoms and its involvement in many fundamental pathways has captivated the interest of scientists for several decades. More recently, after uncovering its potential benefit for human health, the food industry and plant breeders are devoting efforts and resources to improving the GABA levels in food matrices and crops. In plant breeding, NPBTs have demonstrated a higher efficiency and precision than classical crossbreeding for achieving this goal, and studies using induced mutagenesis unlabeled the genetic basis of GABA regulatory pathways, some of which also lead to an increased GABA content in some cases. Nevertheless, large room for improvement exists that can be harnessed *via* the new generation of NPBTs that are emerging at an incredible speed. The progress achieved in tomato could potentially transfer to other crops, taking advantage of the knowledge generated to shorten GABA breeding programs.

## Author Contributions 

Idea conceptualization: HE. Writing—original draft preparation: PG. Writing—review and editing: PG, MT and HE.

## Funding

PG is grateful to the Japanese Society for the Promotion of Science for the JSPS postdoctoral grant FY2019-P19105.

## Conflict of Interest

The authors declare that the research was conducted in the absence of any commercial or financial relationships that could be construed as a potential conflict of interest.
